# Clinical significance of respiratory virus coinfection in children with *Mycoplasma pneumoniae* pneumonia

**DOI:** 10.1186/s12890-022-02005-y

**Published:** 2022-05-30

**Authors:** Soojeong Choo, Yun Young Lee, Eun Lee

**Affiliations:** 1 Department of Pediatrics, Chonnam National University Hospital, Chonnam National University Medical School, Jebong-ro, Dong-gu, Gwangju, 61469 Republic of Korea; 2 Department of Radiology, Chonnam National University Hospital, Chonnam National University Medical School, Gwangju, Republic of Korea

**Keywords:** Children, Coinfection, *Mycoplasma pneumoniae*, Pneumonia, Respiratory virus

## Abstract

**Background:**

The prevalence of refractory *Mycoplasma pneumoniae* (MP) pneumonia has been increasing. However, few studies have investigated the impact of respiratory virus coinfection in patients with MP pneumonia, and their results have been inconclusive. This study aimed to investigate the impact of respiratory virus coinfection in children hospitalized with MP pneumonia.

**Methods:**

This study enrolled 145 children hospitalized with MP pneumonia between May 2019 and March 2020. The patients were divided into two groups: the respiratory virus coinfection and non-coinfection groups. All the children underwent polymerase chain reaction testing for respiratory virus infection. Information on clinical, laboratory, and radiologic findings were obtained retrospectively via medical chart reviews.

**Results:**

Children in the respiratory virus coinfection group were younger than those in the non-coinfection group. Respiratory virus coinfection in children hospitalized with MP pneumonia was significantly associated with persistence of fever more than 6 days (adjusted odds ratio [aOR], 2.394; 95% confidence interval [95% CI], 1.172–4.892**)**, severe pneumonia (aOR, 4.602; 95% CI, 1.154–18.353), and poor response to the stepwise approach for MP pneumonia (aOR, 4.354; 95% CI, 1.374–13.800). In addition, higher levels of liver enzymes and lactate dehydrogenase at admission were associated with respiratory virus coinfection in children with MP pneumonia.

**Conclusions:**

The results of this study suggest that respiratory virus coinfection in children hospitalized with MP pneumonia may be associated with refractory MP pneumonia.

## Background

The hospitalization due to community-acquired pneumonia has been a global big social burden, especially in children [1–3]. Among the diverse pathogens causing lower respiratory infections, *Mycoplasma pneumoniae* (MP) is one of the most common causes of community-acquired pneumonia requiring hospitalization in children [2, 4], accounting for approximately 7–10% of cases, with differences depending on the geographic regions [1, 2, 5]. MP infection has often been considered a self-limiting disease that can be effectively treated using macrolides [6]. However, emergence of macrolide-resistant MP and refractory MP pneumonia has aroused interest due to their influence on the clinical course and therapeutic plan in MP pneumonia [6]. Furthermore, the prevalence of refractory MP pneumonia, ranging from approximately 12–23% [7, 8], has been increasing with that of the macrolide-resistant MP infection, ranging over 80% in South-East Asia [6].

Previous studies have identified predictive biomarkers of refractory MP pneumonia and severe MP pneumonia, such as serum lactate dehydrogenase (LDH) and interleukin 17 A [9–11]. Although macrolide resistance of MP has been a major concern in therapeutic planning for MP infection, recent studies have revealed that macrolide resistance of MP in itself might not be associated with refractory MP pneumonia [12]. Bacterial and viral coinfections in children with community-acquired pneumonia are common, ranging approximately 7% [1]; however, few studies have investigated the influence of respiratory virus coinfection on the clinical course of MP pneumonia and their results have been inconclusive [13–15]. Therefore, in the present study, we aimed to investigate the effects of respiratory virus coinfection on MP pneumonia in children.

## Methods

### Study population

This study enrolled 145 children (mean age ± standard deviation [SD], 6.0 ± 3.8 years; range 0–17 years) hospitalized with MP pneumonia who underwent polymerase chain reaction (PCR) for respiratory virus coinfection, which is routinely performed for all patients hospitalized due to pneumonia in our hospital, from May 2019 to March 2020. The patients were divided into two groups according to the presence of respiratory virus coinfection: the respiratory coinfection and non-coinfection groups. Information on clinical, laboratory, and radiologic findings were obtained retrospectively via medical chart reviews. Our Institutional Review Board (IRB) approved this study and waived the need for informed consent (IRB no. CNUH-2019-261).

### Diagnosis of MP infection

MP infection was confirmed using both PCR (*M. pneumoniae* Real-Time PCR kit, Slan; Biocore, Seoul, South Korea) from sputum or nasopharyngeal swab samples and MP serologic tests (Chorus MP IgM ELISA, Diesse Diagnostica, Senese, Siena, Italy). All the 145 children showed positive PCR results for MP and 108 showed a highly positive MP-specific IgM titer at the time of admission. The remaining 37 children, who were an initially negative for MP-specific IgM, were identified as undergoing seroconversion of MP-specific IgM within one week of hospitalization.

### Definition

All of the children hospitalized with MP pneumonia were treated using a stepwise approach [3], with consideration on the medication administered before admission in our hospital. Among the 145 patients, 97.2% were referred to our hospital because of poor clinical courses. Children hospitalized with MP pneumonia were initially treated with azithromycin for 3 days (10 mg/kg/day, once daily, orally), and those with severe MP pneumonia were treated with intravenous methylprednisolone (1–2 mg/kg/day; maximum 30 mg/dose) [3]. If the patients showed no improvement or progression of MP pneumonia, ciprofloxacin or tetracycline was added in cases of macrolide resistant MP pneumonia. If the patients did not show any improvement within 1 week despite stepwise treatment of MP pneumonia, methylprednisolone (10–15 mg/kg/day) pulse therapy was administered for 3 consecutive days [3].

Response to treatment was classified into three groups: good response, slow response, and no/poor response. A good response was defined as an improvement in respiratory symptoms and/or chest radiography findings within 2–3 days of applying the stepwise treatment for MP pneumonia after hospitalization; a slow response was defined as a slight improvement in respiratory symptoms and/or chest radiography findings within 1 week, but not within 2–3 days, with applying the stepwise treatment for MP pneumonia; and no/poor response or progression was defined as the absence of any improvement in the progression of respiratory symptoms and/or chest radiography findings even after 1 week of applying the stepwise treatment for MP pneumonia [16].

The severity of pneumonia based on the extent of pneumonic infiltration on the chest radiography at the time of admission was defined as follows: mild, pneumonic lesions involving < 1/3 of the total lung volume; moderate, pneumonic lesion involving more than 1/3, but less than 1/2, of the total lung volume; and severe, pneumonic lesions involving more than 1/2 of the total lung volume. Post-infectious bronchiolitis obliterans was diagnosed based on a combination of medical history, clinical features, and chest high-resolution computed tomography findings, including mosaic ground-glass patterns, air-trapping, and/or bronchial thickening [17–19]. Pulmonary thromboembolism was diagnosed based on the computed tomography angiography findings, including the partial filling defects [20].

### Microbiological studies

The cut-off values for interpreting an MP infection status based on the MP-specific IgM titers were assessed in accordance with the manufacturer’s instructions, as positive (IgM, > 1.1) and negative (IgM, < 0.9). Macrolide resistance was evaluated by identifying the point mutations at both positions 2063 and 2064 in domain V of the 23 S rRNA of MP by the GENECUBE system and GENECUBE *Mycoplasma* detection kit (Sin Corporation, Tokyo, Japan). The presence of 16 respiratory viral pathogens, including adenovirus, respiratory syncytial viruses A and B, rhinovirus, influenza viruses A and B, parainfluenza viruses 1–4, bocavirus, metapneumovirus, enterovirus, and corona viruses OC43, 229E, and NL63, was detected in nasopharyngeal swab samples by means of a PCR assay using the Anyplex II RV16 Detection kit (Seegene, Seoul, South Korea). In some of the patients with available sputum samples, the Pneumobacter PCR (Allplex Pneumobacter Assay, Seegene, Seoul, South Korea) assay was performed, which can detect *Streptococcus pneumoniae*, *Haemophilus influenza*, MP, *Chlamydophila pneumoniae*, *Bordetella pertussis*, and *Legionella pneumophila*.

### Statistical analysis

Comparisons of demographic characteristics, clinical courses, laboratory findings, microbiological features, and radiologic findings between the non-coinfection and coinfection groups were performed using chi-square tests as appropriate for the variables. Logistic regression analysis was performed to identify the factors associated with respiratory virus coinfection in patients with MP pneumonia, after adjustment for age and sex. To exclude the effect of bacterial coinfection, adjustment for age, sex, and bacterial coinfection was also performed. All statistical analyses were performed using IBM SPSS Statistics for Version 24.0 (IBM Corp., Armonk, NY, USA). A *P* value < 0.05 was considered significant.

## Results

### Demographic characteristics of the study population

**Fig. 1:**
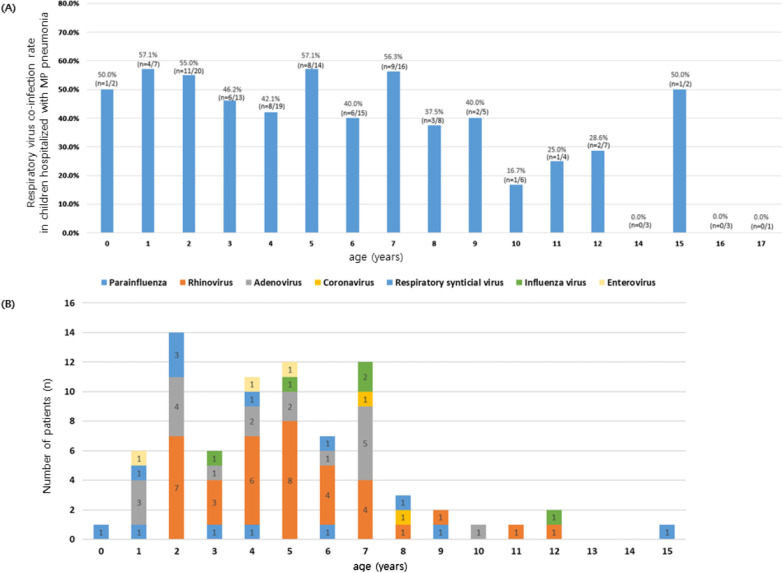
Respiratory virus co-infection rate in children hospitalized with *Mycoplasma pneumoniae* (MP) pneumonia according to age (**A**). Distribution of co-infected respiratory viruses according to age in children hospitalized with *Mycoplasma pneumoniae* pneumonia (**B**)

The demographic characteristics of the study population are described in Table 1 and the respiratory virus co-infection patterns in children hospitalized with MP pneumonia are shown in Fig. 1. In the total population hospitalized with MP pneumonia, 43.4% had respiratory virus coinfection. Children in the respiratory virus coinfection group were significantly younger than those in the non-coinfection group (mean ± SD, 5.0 ± 3.1 vs. 6.7 ± 4.2 years, *P* = 0.008; Table 1). The proportion of asthma history was higher in the respiratory virus coinfection group than in the non-coinfection group, but the difference was not statistically significant (17.5% vs. 7.3%, *P* = 0.060). The most commonly co-infected respiratory virus was rhinovirus (36/63, 57.1%, Table 2), followed by adenovirus (19/63, 30.2%).

**Table 1:** Comparison of the baseline and clinical characteristics according to the presence of respiratory virus co-infection

Variable, n (%) or mean ± SD	Respiratory virus non-coinfection group	Respiratory virus coinfection group	*P* value
*Baseline characteristics*			
N (%)	82 (56.6)	63 (43.4)	NA
Age at the diagnosis of MP pneumonia, mean ± SD, years	6.7 ± 4.2	5.0 ± 3.1	**0.008**
Male, n (%)	40/42 (48.8)	35/63 (46.7)	0.418
Referred cases, n (%)	80/82 (97.6)	61/63 (96.8)	0.789
Comorbid allergic diseases			
Atopic dermatitis	1/82 (1.2)	2/63 (3.2)	0.412
Allergic rhinitis	39/82 (47.6)	38/63 (60.3)	0.127
Asthma	6/82 (7.3)	11/63 (17.5)	0.060
*Clinical characteristics*			
Duration between symptom onset and admission, days	5.7 ± 3.1	7.9 ± 4.3	**0.001**
Duration of fever during illness, days	5.7 ± 3.8	7.7 ± 4.7	**0.006**
Hemoptysis	3/82 (3.7)	0/63 (0.0)	0.125
Fever	81/82 (98.8)	63/63 (100.0)	0.379
Oxygen supplementation	6/82 (7.3)	3/63 (4.8)	0.527
ICU admission	0/82	0/63	NA
Response to treatment of MP pneumonia			**0.019**
Good response	38/82 (46.3)	16/62 (25.8)	
Slow response	38/82 (46.3)	35/62 (56.5)	
No/poor response	6/82 (7.3)	11/62 (17.7)	
Total duration of hospitalization, days	8.0 ± 4.3	9.8 ± 5.2	**0.033**

Bold values denote statistical significance

*ICU* intensive care unit, *MP* *Mycoplasma pneumoniae*, *NA* not applicable, *SD* standard deviation

**Table 2:** Co-infected respiratory virus in MP pneumonia in children

Respiratory virus	n (%)*
Rhinovirus	36/63 (57.1)
Adenovirus	19/63 (30.2)
Respiratory syncytial virus	7/63 (11.1)
Parainfluenza virus	7/63 (11.1)
Corona virus	2/63 (3.2)
Influenza virus	5/63 (7.9)
Enterovirus	3/63 (4.8)

*When patients were co-infected with more than two respiratory viruses, each respiratory virus was independently counted

*MP* *Mycoplasma pneumoniae*

### Comparison of clinical manifestations between the respiratory virus coinfection and non-coinfection groups

The total durations of fever during the illness (mean ± SD, 7.7 ± 4.7 vs. 5.7 ± 3.8 days, *P* = 0.006) and hospitalization (mean ± SD, 9.8 ± 5.2 vs. 8.0 ± 4.3 days, *P* = 0.033) were longer in the respiratory virus coinfection group than in the non-coinfection group (Table 1). The response to the stepwise treatment for MP pneumonia was also poorer in the respiratory virus coinfection group than in the non-coinfection group (*P* = 0.019, Table 1).

### Comparison of laboratory findings between the respiratory virus coinfection and non-coinfection groups

The white blood cell counts, levels of LDH, aspartate aminotransferase (AST), alanine aminotransferase (ALT), and MP-specific IgM titer were significantly higher in the respiratory virus coinfection group than in the non-coinfection group (Table 3).

**Table 3:** Comparisons of laboratory findings and microbiological characteristics according to the presence of respiratory virus co-infection

Variable, mean ± SD (range)	Respiratory virus non-coinfection group	Respiratory virus coinfection group	*P* value
Laboratory findings			
WBC, × 10^3^/µL	8500 ± 4200	10,300 ± 5100	**0.026**
Neutrophil (%)	63.5 ± 13.1	62.7 ± 16.8	0.764
Lymphocyte (%)	25.8 ± 11.1	25.0 ± 13.1	0.669
Eosinophil (%)	2.0 ± 2.4	1.6 ± 2.5	0.292
Monocyte (%)	8.4 ± 3.9	8.5 ± 3.7	0.832
CRP, mg/dL	3.8 ± 5.1	2.6 ± 4.2	0.123
ESR, mm/h	38.8 ± 20.1	33.2 ± 19.3	0.141
Procalcitonin, ng/dL	0.3 ± 0.4	0.3 ± 0.5	0.969
LDH, IU/L	724.1 ± 318.1	905.8 ± 357.3	**0.002**
AST, IU/L	37.8 ± 32.1	53.0 ± 37.0	**0.011**
ALT, IU/L	26.3 ± 27.2	41.1 ± 47.0	**0.029**
Albumin, g/dL	5.3 ± 1.5	5.3 ± 1.5	0.848
MP specific IgM titer at admission, index	3.8 ± 3.1	5.0 ± 3.2	**0.025**
Microbiological characteristics			
Macrolide sensitivity			0.714
MSMP	5/81 (6.2)	3/63 (4.8)	
RMP	76/81 (93.8)	60/63 (95.2)	
Bacterial co-infection identified using Pneumobacter PCR	12/70 (17.1)	15/59 (25.4)	0.249
Adenovirus infection	NA	19/63 (30.2%)	NA
Rhinovirus infection	NA	36/63 (57.1%)	NA

Bold values denote statistical significance

*AST* aspartate aminotransferase, *ALT* alanine aminotransferase, *CRP* C-reactive protein, *ESR* erythrocyte sedimentation rate, *IgM* immunoglobulin M, *LDH* lactate dehydrogenase, *MP* *Mycoplasma pneumoniae*, *MRMP* macrolide-resistant *Mycoplasma pneumoniae*, *MSMP* macrolide-sensitive *Mycoplasma pneumoniae*, *PCR* polymerase chain reaction, *SD* standard deviation, *WBC* white blood cell

### Comparison of microbiological and radiologic characteristics between the respiratory virus coinfection and non-coinfection groups

Among the 27 patients with positive results in the Pneumobacter PCR test, *Streptococcus pneumoniae* was found in 8 cases; *Haemophilus influenzae*, in 11 cases; and both *S. pneumoniae* and *H. influenzae* in 8 cases. No significant differences were observed in the proportion of macrolide resistance in MP pneumonia and bacterial coinfection between the respiratory virus coinfection and non-coinfection groups (Table 3). In addition, no significant differences were observed in chest radiography findings at admission and the proportion of pleural effusion (Table 4).

**Table 4:** Comparison of radiologic findings according to the presence of respiratory virus co-infection

Variables, n (%)	Respiratory virus non-coinfection group	Respiratory virus coinfection group	*P* value
Severity of pneumonia at admission based on chest radiograph findings			0.225
Mild	11 (13.4)	4 (6.3)	
Moderate	55 (67.1)	41 (65.1)	
Severe	16 (19.5)	18 (28.6)	
Chest radiography findings at admission			0.480
Lobar consolidation	21 (25.6)	14 (22.2)	
Patchy consolidation	39 (47.6)	24 (38.1)	
Peribronchial infiltration	17 (20.7)	19 (30.2)	
Diffuse nodular opacity	2 (2.4)	1 (1.6)	
Diffuse infiltration	3 (3.7)	5 (7.9)	
Pleural effusion	11/82 (13.4)	12/63 (19.0)	0.357
Development of PTE	1/82 (1.2)	3/63 (4.8)	0.197
Development of PIBO	4/82 (4.9)	12/63 (19.0)	**0.007**

Bold values denote statistical significance

*PIBO* post-infectious bronchiolitis obliterans, *PTE* pulmonary thromboembolism

### Factors associated with respiratory virus coinfection in children with MP pneumonia

Respiratory virus coinfection in children hospitalized with MP pneumonia was associated with younger age (adjusted odds ratio [aOR], 0.879; 95% confidence interval [CI] 0.798–0.969), persistence of fever more than 6 days (aOR, 2.394; 95% CI 1.172–4.892), severe pneumonia based on chest radiography findings (aOR, 4.602; 95% CI 1.154–18.353), and poor response to the stepwise treatment for MP pneumonia (slow response, aOR 2.187, 95% CI 1.041–4.599; moderate response, aOR 4.354, 95% CI 1.374–13.800; Table 5), after adjustment for age and sex. In addition, increased levels of LDH and abnormal liver function, as indicated by elevated levels of AST and ALT, were associated with respiratory virus coinfection in children hospitalized with MP pneumonia. On adjustment for age, sex, and bacterial coinfection, the similar results were observed, except for severe pneumonia and slow response to the treatment for MP pneumonia (Table 5).

**Table 5:** Factors associated with respiratory virus co-infection in children with MP pneumonia

Variables	OR	*P* value	aOR*	*P* value	aOR**	*P* value
Age at diagnosis of pneumonia, years	**0.881 (0.800–0.970)**	**0.010**	**0.879 (0.798–0.969)**	**0.009**	**0.898 (0.809–0.997)**	**0.045**
^†^Duration of fever during illness ≥ 6 days	**2.105 (1.074–4.123)**	**0.030**	**2.394 (1.172–4.892)**	**0.017**	**3.329 (1.540–7.196)**	**0.002**
Total duration of hospitalization, days	**1.083 (1.005–1.167)**	**0.037**	1.070 (0.992–1.154)	0.080	1.060 (0.979–1.147)	0.152
Severity of pneumonia based on chest radiography findings at admission						
Mild	Ref.		Ref.		Ref.	
Moderate	2.050 (0.609-6.900)	0.246	2.746 (0.787–9.578)	0.113	2.485 (0.589–10.490)	0.215
Severe	3.094 (0.820-11.672)	0.096	**4.602 (1.154–18.353)**	**0.031**	4.184 (0.875–20.011)	0.073
WBC, x 10^3^/µL	**1.085 (1.008–1.168)**	**0.030**	1.065 (0.988–1.148)	0.102	1.053 (0.974–1.139)	0.193
AST ≥ 44 IU/L^†^	**3.300 (1.577–6.906)**	**0.002**	**3.491 (1.616–7.543)**	**0.001**	**5.202 (2.211–12.238)**	**< 0.001**
ALT ≥ 33 IU/L^†^	**2.749 (1.290–5.856)**	**0.009**	**3.325 (1.478–7.477)**	**0.004**	**4.756 (1.903–11.887)**	**0.001**
LDH ≥ 805.3 IU/L^†^	**6.250 (2.878–13.574)**	**< 0.001**	**5.704 (2.602–12.504)**	**< 0.001**	**6.555 (2.772–15.503)**	**< 0.001**
CRP, mg/dL	0.939 (0.866–1.019)	0.132	0.972 (0.898–1.051)	0.471	0.960 (0.885–1.042)	0.333
MP-specific IgM titer at the time of admission, index	**1.129 (1.014–1.257)**	**0.026**	1.109 (0.993–1.239)	0.065	1.121 (0.995–1.263)	0.061
Response to treatment						
Good response	Ref.		Ref.		Ref.	
Slow response	**2.187 (1.041–4.599)**	**0.039**	**2.187 (1.041–4.599)**	**0.039**	2.197 (0.931–5.185)	0.072
No/poor response	**4.354 (1.374–13.800)**	**0.012**	**4.354 (1.374–13.800)**	**0.012**	**4.410 (1.273–15.277)**	**0.019**

Bold values denote statistical significance

*aOR* adjusted odds ratio, *AST* aspartate aminotransferase, *ALT* alanine aminotransferase, *CRP* C-reactive protein, *IgM* immunoglobulin M, *LDH* lactate dehydrogenase, *MP* *Mycoplasma pneumoniae*, *NA* not applicable, *PCR* polymerase chain reaction, *Ref.* reference

*Adjusted by age and sex

**Adjusted by age, sex, and bacterial coinfection identified using Pneumobacter PCR or respiratory culture from sputum samples

^†^The variables were dichotomized based on the mean values

## Discussion

In this study, we identified that a longer duration of fever, severe pneumonia, and poor response to the stepwise treatment for MP pneumonia were associated with respiratory virus coinfection in children hospitalized with MP pneumonia. These results suggest that respiratory virus coinfection might be a contributing factor for refractory MP pneumonia in children. In addition, the levels of liver enzymes, including AST and ALT, and LDH were higher in the respiratory virus coinfection group than in the non-coinfection group. This finding supports a higher disease burden in the respiratory virus coinfection group than in the non-coinfection group. These results highlight the importance of respiratory virus coinfection in children with MP pneumonia and therefore, might be applied for therapeutic planning and prognosis prediction in children with MP pneumonia, especially in the era of an increasing prevalence of refractory MP pneumonia cases.

Although the respiratory virus coinfection rates in children with MP pneumonia are known to vary according to study population, the present study showed that the respiratory virus coinfection in children with MP pneumonia is common (43.4%). Previous studies have shown that the respiratory virus coinfection rates in children with MP pneumonia ranged from 27.3 to 56.1% [13, 14, 21]. Moreover, respiratory virus coinfection in MP pneumonia was more commonly observed in younger children (Fig. 1), consistent with the results of previous studies [1, 4].

Respiratory syncytial virus is the most common cause of community-acquired pneumonia in children across all ages, followed by rhinovirus [2]. In this study, rhinovirus was the most common cause of respiratory virus coinfection in children with MP pneumonia (57.1%, n = 36/63; Table 2; Fig. 1); this finding was consistent with that of a previous study (20/30, 66.7%) [13]. These findings suggest that the commonly infected respiratory virus is also the main cause of respiratory virus coinfection even in MP pneumonia in children. We found that the respiratory virus coinfection may affect the clinical course of MP pneumonia, although the respiratory virus in itself does not generally cause serious respiratory tract infections in healthy children [22].

In the present study, we observed significant associations of respiratory virus coinfections with severe clinical manifestations of MP pneumonia and poor response to the stepwise treatment for MP pneumonia. These findings suggest that a respiratory virus coinfection in MP pneumonia may be associated with more severe clinical outcomes than those in pneumonia caused by a viral infection alone. Studies on the mechanisms underlying the effects of respiratory virus coinfections in MP pneumonia are currently lacking. The altered immune function during the illness of MP pneumonia or immunomodulatory drugs, such as systemic corticosteroids, commonly used for the treatment of MP pneumonia, may play a role in the association of poor clinical outcomes with respiratory virus coinfections in MP pneumonia [23, 24]. Although corticosteroids are commonly used as adjuvant therapy in viral pneumonia, acute respiratory distress syndrome, or severe pneumonia due to their anti-inflammatory effects [25], the effects of corticosteroids on respiratory diseases can differ according to the severity of diseases, degree of inflammation, and immune status of hosts [25]. Therefore, these factors must be considered when administrating systemic corticosteroids in patients with MP pneumonia, especially when the patients are coinfected with respiratory viruses.

A previous study showed that the duration of fever was longer in children with MP pneumonia and respiratory viral coinfection than in those without respiratory virus coinfection. However, that study showed no significant differences in clinical features and severity of pneumonia between the two groups [13]. Another study reported no significant differences in clinical characteristics and outcomes according to the presence of respiratory virus coinfection in MP pneumonia [14]. However, the present study showed that respiratory virus coinfection was associated with refractory MP pneumonia, as reflected by severe pneumonia and poor response to active stepwise treatment for MP pneumonia among children hospitalized with MP pneumonia with respiratory virus coinfection. Consistent results were obtained after adjustment for bacterial coinfection (Table 5). Therefore, the present results can be applied when treating patients in the tertiary hospital setting, as is reflected by the high referral rate (97.2%). Although the differences in clinical characteristics and clinical outcomes among patients with MP pneumonia and respiratory virus coinfection across studies might be partially attributable to the differences in the characteristics of the study populations, the severity of pneumonia in the enrolled patients, or microbiological characteristics, such as the serotypes of respiratory pathogens, the results of the present study are important because the results of the present study clearly indicate that respiratory virus coinfection can be a causative factor for refractory MP pneumonia in children. These findings may aid in the development of therapeutic strategies for refractory MP pneumonia.

The asymptomatic elevation of liver enzyme levels is frequently encountered in MP infection [26]. The present study showed that the asymptomatic elevation of liver enzyme levels was more prominent in children with MP pneumonia with respiratory virus coinfection than in those without coinfection. Considering that elevated liver enzyme levels are more commonly observed in respiratory virus infections at a younger age and that respiratory virus coinfection is more common at a younger age [27], the higher prevalence of respiratory virus coinfection in younger children might be associated with significantly higher levels of liver enzymes in the respiratory virus coinfection group. However, the clinical importance of elevated liver enzyme levels in the present study was weak because the abnormal levels of liver enzymes were asymptomatic and these levels normalized during the period of illness.

MP infection can be confirmed using PCR and/or serology tests, although both tests have limitations in the early diagnosis of MP infection [28]. A negative MP-specific IgM titer, especially in the early phase of illness, and false negative results of PCR, partially due to poor cooperation during sample collection and the sampling site, can lead to a missed diagnosis of MP infection [29, 30]. In addition, PCR and MP-specific IgG tests can cause misdiagnosis of a current MP infection due to their long-term positivity [31]. Therefore, we confirmed MP infection using both a PCR assay and MP-specific IgM titer, especially a high titer, combined with medical history, symptoms, physical examination, and chest radiographic findings [29, 30].

This study has several limitations. Most of the patients were referred, and therefore, the treatment strategies for MP pneumonia before referral to our hospital were different and might have affected the clinical course of MP pneumonia. To address this issue, we treated all patients hospitalized with MP pneumonia using the stepwise treatment approach for MP pneumonia after considering the treatment received at previous hospitals [3]. The generalizability of the present results is limited because this study enrolled referred children with uncontrolled or persistent MP pneumonia. However, since refractory MP pneumonia is more commonly observed in severe cases of MP pneumonia, the present findings are still meaningful. Although most cases of MP infection in the present study involved MRMP, macrolides were used as the first step in the stepwise treatment of MP pneumonia due to the clinical effectiveness of macrolides even in some cases of MRMP infection [32–34] and because of concerns regarding the safety and potential development of resistance to the second-line therapy. This approach also allowed us to perform tests to identify macrolide resistance of MP.

## Conclusions

In conclusion, we identified the clinical significance of respiratory virus coinfection in children with MP pneumonia. Our results suggest that respiratory virus coinfection in children hospitalized with MP pneumonia may be associated with refractory MP pneumonia. Respiratory virus coinfection should be considered in children with MP pneumonia showing poor or no response to the stepwise treatment approach.

## Data Availability

Data are available from the corresponding author upon reasonable request.

## Abbreviations

aOR: Adjusted odds ratio
AST: Aspartate aminotransferase
ALT: Alanine aminotransferase
CRP: C-reactive protein
IgM: Immunoglobulin M
LDH: Lactate dehydrogenase
MP: *Mycoplasma pneumoniae*
NA: Not applicable
PCR: Polymerase chain reaction
Ref.: Reference
